# Role of Preoperative Multiple-Drug-Resistant Bacteria Intestinal Colonization in Cardiac Surgery: A Retrospective Study

**DOI:** 10.3390/jcm13226897

**Published:** 2024-11-16

**Authors:** Alessia Mattei, Martina Cuccarelli, Lorenzo Schiavoni, Antonio Nenna, Giuseppe Pascarella, Alessandro Ruggiero, Lelio Carpinteri, Fabio Costa, Mario Lusini, Ciro Mastroianni, Raffaele Barbato, Massimo Chello, Massimiliano Carassiti, Rita Cataldo, Felice Eugenio Agrò, Alessandro Strumia

**Affiliations:** 1 Operative Research Unit of Anesthesia and Intensive Care, Fondazione Policlinico Universitario Campus Bio-Medico, 00127 Roma, Italy; a.mattei@policlinicocampus.it (A.M.); g.pascarella@policlinicocampus.it (G.P.); f.costa@policlinicocampus.it (F.C.); m.carassiti@policlinicocampus.it (M.C.); r.cataldo@policlinicocampus.it (R.C.); f.agro@policlinicocampus.it (F.E.A.); a.strumia@policlinicocampus.it (A.S.); 2 Research Unit of Anaesthesia and Intensive Care, Department of Medicine, University Campus Bio-Medico di Roma, 00128 Roma, Italy; martina.cuccarelli@unicampus.it (M.C.); alessandro.ruggiero@unicampus.it (A.R.); 3 Cardiac Surgery Unit, Fondazione Policlinico Universitario Campus Bio-Medico, 00198 Rome, Italy; a.nenna@policlinicocampus.it (A.N.); m.lusini@policlinicocampus.it (M.L.); c.mastroianni@policlinicocampus.it (C.M.); r.barbato@policlinicocampus.it (R.B.); m.chello@policlinicocampus.it (M.C.); 4 Department of Anesthesia and Intensive Care, Fondazione Policlinico Universitario Agostino Gemelli IRCCS, Università Cattolica del Sacro Cuore, 00168 Roma, Italy; lelio.carpinteri@guest.policlinicogemelli.it

**Keywords:** MDR infections, cardiac surgery, mortality, rectal swab

## Abstract

**Background/Objectives:** Multiple-drug-resistant (MDR) bacteria are pathogens resistant to three or more antibiotic classes, and infection with these bacteria is associated with increased mortality, morbidity, and hospital management costs. Given the rise in antibiotic resistance, selecting appropriate antimicrobial drugs and avoiding the unnecessary use of new antibiotics are crucial. Due to their nosocomial nature, monitoring and preventing MDR infections are essential. **Methods:** This study enrolled patients who underwent cardiac surgery from January 2020 to May 2022. The patients included were those 18 years or older who tested positive for MDR intestinal colonization before surgery. Excluded were patients who tested positive after surgery, or were younger than 18, pregnant, or lactating. For each positive patient, the National Surgical Quality Improvement Program (NSQIP) score was calculated. The data collected included age, gender, body mass index (BMI), the type of surgery, the intensive care unit (ICU) length of stay, ICU readmission, mortality, and other infections (pneumonia, bacteremia, or surgical site infection) to establish a control group and postoperative outcome measures. **Results:** No statistically significant differences were found between the groups regarding the ICU length of stay, new ICU admissions, or mortality. Additionally, there were no differences in the infection rates, such as bacteremia, wound infections, and pneumonia. **Conclusions:** Preoperative rectal colonization by MDR bacteria does not appear to worsen postoperative outcomes for cardiac surgery patients. These findings suggest that patients with a preoperative MDR-positive rectal sample might undergo cardiac surgery without significantly increased risk. Besides the limitations of this study, pre-surgical antibiotic prophylaxis may not need to be adjusted for patients with preoperative MDR positivity.

## 1. Introduction

Infections caused by multidrug-resistant bacteria are a significant concern for health services worldwide [1]. Multidrug-resistant (MDR) bacteria consist of pathogens that can evade the effects of three or more antibiotic classes [2]. In this regard, physicians must carefully select the appropriate antibiotic therapy, relying on antibiograms for guidance [3]. Considering the emerging phenomenon of antibiotic resistance, it is also crucial to make the right choice, avoiding the use of the newest and most powerful available drugs unless strictly necessary.

Therefore, monitoring and preventing MDR organism (MDRO) infections are crucial strategies to fight them effectively [4]. To accomplish these tasks, the patients who are most likely to develop an MDR infection should be identified as soon as possible. However, there is a significant lack of agreement regarding both the impact and the management of MDR colonization, especially in patients undergoing invasive procedures and surgery.

Cardiac surgery patients, overall, are often fragile and affected by multiple comorbidities, which, combined with the invasiveness of the procedures, can negatively impact the postoperative course. Additionally, the use of cardiopulmonary bypass can facilitate systemic infections through various mechanisms, such as the translocation of enteric bacteria [5,6].

To date, only a limited number of studies have been performed in this field, with only two studies focusing on other types of surgery, such as biliopancreatic surgery. Moreover, two different research groups have noticed an increase in mortality and morbidity in hepatic [7] and pancreatic surgery [8], but their results differed from each other.

Recently, an attempt to define guidelines for MDR colonization management has been made. However, the authors of these guidelines acknowledge that the levels of recommendation and evidence must be considered as generally low [9].

Although the overall burden of postoperative MDR bacterial infections in adult patients undergoing invasive cardiovascular procedures is significant [10], only a few studies have investigated the role of previous MDR colonization as a preoperative risk predictor in this field [11,12,13].

In our study, we aimed to retrospectively analyze the role of preoperative MDR intestinal colonization in terms of postoperative outcomes for MDR infection in adult patients after cardiac surgery.

## 2. Materials and Methods

In this study, patients who underwent cardiac surgery between January 2020 and May 2022 were reviewed for MDR rectal colonization positivity. The study was approved by the Fondazione Policlinico Campus Bio-Medico’s ethics committee in March 2023 with the number PAR 20.23 OSS. All the enrolled patients provided informed consent and agreed to participate in this research.

Initially, data from 27 patients who tested positive for MDR rectal colonization by Klebsiella pneumoniae, vancomycin-resistant Enterococci, Acinetobacter baumannii, or Pseudomonas aeruginosa were collected and recorded in specific databases.

We included the patients who tested positive for MDR intestinal colonization before cardiac surgery and who were 18 years old or older. The exclusion criteria were individuals younger than 18 years old and patients who declined to participate.

Our hospital management for MDR patients includes the early identification of colonization or infection through routine screening (e.g., rectal swabs) at admission, especially when patients are transferred from other facilities or hospitalized for at least one week. Strict contact precautions are implemented in the case of a positive result, and the colonized patients are isolated in single rooms when possible. It is noteworthy that the prevalence of MDR-colonized patients in our ICU is approximately 10%, which aligns with the data reported by other centers in the literature [14,15].

All the patients received prophylactic cefazolin, with 2 g administered preoperatively, 1 g after weaning from extracorporeal circulation, and then 1 g every 6 h for the first 24 h postoperatively, as per our hospital protocol for cardiac surgery patients. In cases of cephalosporin allergy, 600 mg of clindamycin was given preoperatively and then every 12 h for the same duration.

Age, gender, BMI, and the type of surgery were used as baseline characteristics to form a control group through propensity score matching. This anonymous and retrospective group consisted of the patients who underwent cardiac surgery at our hospital during the same period.

For each patient enrolled in the study group (those with a positive rectal swab), the National Surgical Quality Improvement Program (NSQIP) score, which typically assesses the risk of perioperative mortality and morbidity based on medical history and surgical complexity, was calculated. The score incorporates patient-specific factors such as age, comorbidities, functional status, and the type of surgical procedure to provide an individualized risk assessment. It is derived from a large, multi-institutional database of clinical outcomes, allowing it to predict risks for a wide variety of surgeries.

The same medical history data and NSQIP score were collected for the control group, ensuring comparability with the patients who tested positive.

The primary endpoint of this study was to investigate the development of infections after surgery, specifically identified as bacteremia, pneumonia, and surgical site infection. The secondary endpoints regarded differences in the ICU length of stay, new ICU admissions, and mortality. The data collected about the patients included age, gender, body mass index (BMI), the type of surgery (coronary artery bypass graft surgery, prosthetic heart valve surgery, etc.), the length of stay in the intensive care unit (ICU), ICU readmission, mortality, and the development of infections such as pneumonia, bacteremia, or surgical site and wound infections.

The follow-up was set at 30 days after surgery.

**Figure 1:**
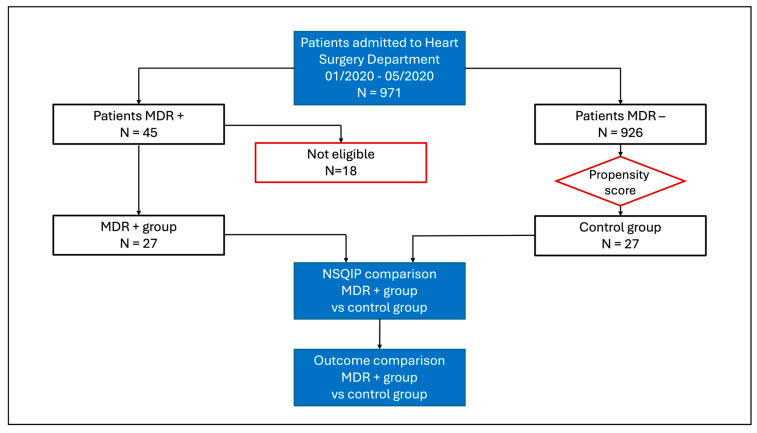
Flow chart.

Figure 1 shows the study flow chart.

## 3. Statistical Analysis

Considering the 27 patients in the MDR+ group eligible for the study, the control group of 926 patients underwent propensity score matching to produce a comparable group of 27 patients per group.

The propensity score matching was performed by means of a probit regression, considering “MDR” (the grouping variable) as the dependent variable. A non-greedy approach to pre-surgical variables was adopted to pair similar patients across both groups. The caliper method, employing a nearest neighbor algorithm (1:1) without repetition, was applied to minimize assignment bias from confounding variables.

The variables included in the propensity score were age, sex, BMI, the type of surgery (CABG vs. other than CABG), and EuroSCORE II (Table 1).

This method ensured that units with similar propensity scores in both the treatment and the control groups helped mitigate potential bias. This model was chosen as it produced the greatest amount of meta-bias reduction, as evaluated with the post-estimation tests of propensity score matching (Supplementary Table S1). After matching, the comparison of the outcomes between these two groups was performed using both the chi-square test for independent variables and the *t*-test and the Mann–Whitney test for continuous data (based on normality criteria). A two-tailed *p* value < 0.05 was highlighted as statistically significant. The statistical analysis was performed with STATA version 16 (personal license) using the package “psmatch2” and the related functions.

## 4. Results

Fourteen patients with positive rectal swabs underwent CABG surgery, six underwent aortic valve replacement, four underwent mitral valve replacement, two underwent combined aortic and mitral valve replacement, and one underwent combined aortic valve and ascending aorta replacement. In the control group, 11 patients underwent CABG surgery, 7 underwent aortic valve replacement, 5 underwent mitral valve replacement, 2 underwent combined aortic and mitral valve replacement, and 2 underwent combined aortic valve and ascending aorta replacement. All of the patients underwent median sternotomy as the surgical approach and had mediastinal/pleural thoracic drains positioned at the end of the procedure.

A comparison of the preoperative variables used to establish the control group through propensity score matching showed no significant differences. The comparisons between the matched and the unmatched cohorts are reported in Table 2.

Similarly, the preoperative median NSQIP values were comparable between the groups and are presented in Table 3.

No statistical differences were observed in the postoperative infections such as bacteremia (*p* = 0.552), wound infections (*p* = 0.125), and pneumonia (*p* = 0.999).

Numerically, bacteremia occurred in two patients who were preoperatively colonized and in one patient in the control group. Pneumonia was observed once in both groups. Surgical site infections occurred in six colonized patients and in two non-colonized patients.

In the positive swab group, postoperative infections (of any kind) were caused by the same MDR pathogen identified preoperatively in five patients, accounting for 55% of those who developed infections. Specifically, Klebsiella pneumoniae caused infections in three patients, including one case of pneumonia and two cases of bacteremia, and Pseudomonas aeruginosa in two patients, both presenting surgical site infections. In the control group, only one patient developed pneumonia caused by an MDR pathogen (Klebsiella pneumoniae), representing 25% of those who developed infections.

No statistically significant difference was found between the two groups in terms of the ICU length of stay, new ICU admissions, and mortality. The average ICU length of stay was 2 days in both groups (*p* = 0.448). There were numerical differences observed in the ICU readmissions (four cases in the experimental group vs. one case in the control group). However, statistical significance was not reached (*p* > 0.05). Finally, there was one death in each group, both due to cardiac causes and not related to sepsis or any kind of infection. The main outcomes are presented in Table 4.

## 5. Discussion

The current spread of antibiotic-resistant bacteria could severely impact human health. However, there remains significant disagreement regarding both the impact and the management of MDR colonization in patients undergoing invasive surgical procedures. Furthermore, the current medical literature does not provide a definitive answer to a crucial question: Are patients colonized by MDR bacteria at an increased risk of infection after major surgery?

It is widely accepted that preoperative colonization by MDR pathogens may be difficult to eradicate following various surgical procedures. However, what if the use of appropriate antibiotic therapy could suppress the activity of these pathogenic bacteria?

In such cases, meticulous perioperative management—including preoperative contact isolation and the administration of protocol-driven prophylactic antibiotics based on established guidelines—could serve as effective preventive measures. This approach may also improve patient outcomes by reducing the risk of postoperative complications.

The incidence of complications from MDR bacterial infections in patients undergoing all types of cardiac surgery, including open procedures, has been found to range from 0.6% to 10%, with mortality rates significantly higher compared to patients with non-MDR infections [16]. Patients with MDRO infections have longer post-infection hospital stays and overall hospitalization periods, with a numerically higher all-cause mortality rate compared to non-MDRO infection cases [17].

The current medical literature has inadequately explored this area, particularly in cardiac surgery, with the exception of methicillin-resistant S. aureus (MRSA) [11].

To address this critical gap, the primary aim of this study was to investigate the development of infections after surgery, identified as bacteremia, pneumonia, and surgical site infection. Additionally, we investigated the potential changes in the ICU length of stay, ICU readmission, and mortality. We focused on Gram-negative bacteria due to their predominant role in rectal colonization and their association with systemic infections, particularly considering the potential for bacterial translocation to be exacerbated by extracorporeal circulation during surgery [5,6].

To the best of our knowledge, this is the first study to explore the impact of preoperative MDR intestinal colonization in heart surgery patients. One of the few available retrospective studies analyzed the clinical characteristics and risk predictors of MDRO infections in adult patients after cardiac surgery, but it did not include comparisons related to preoperative colonization, which limits the ability to draw conclusions about its direct impact [17].

A univariate analysis identified several risk factors for postoperative MDRO infection, including undergoing coronary artery bypass graft (CABG) and a secondary operation, pre-infection exposure to vancomycin and linezolid, combination antibiotic therapy, the use of four antibiotics in combination, glucocorticoid use, and preoperative hypoalbuminemia. A multivariate regression analysis further revealed that undergoing CABG, a secondary operation, and pre-infection exposure to linezolid were independent risk predictors for MDRO infection. The study also noted that the ICU length of stay and the duration of hospitalization before the infection diagnosis significantly increased the risk of MDRO infection.

While this study identifies multiple risk factors influencing the occurrence of MDRO infections, it does not extensively explore the impact of prolonged postoperative hospitalization on the increased risk of MDRO colonization.

In this study, we evaluated the postoperative outcomes of cardiac surgery patients who tested positive for MDR rectal colonization, comparing them with a negative control group. We found that intestinal colonization did not have a statistically significant impact on the outcomes of the patients undergoing cardiac surgery. The median length of stay was 2 days in both groups. There were no differences in the incidence of pneumonia or mortality during admission, each occurring once in both groups. Statistically insignificant differences were observed in ICU readmission (four cases in the experimental group vs. one case in the control group), bacteremia (two cases in the experimental group vs. one case in the control group), and surgical site infection (six cases in the experimental group vs. two cases in the control group).

It is reasonable that patient colonization may represent a more significant trigger for infection in the presence of other risk factors.

A Brazilian study on MDR bacterial infections indicated that the predominant demographics affected by MDR bacteria were males and hospitalized patients over 60 years of age (55.1%) [18].

Similarly, Lorenzoni et al. [19] demonstrated that the incidence of MDR infections after cardiac surgery is higher among male patients over 60 years old. The common indications for cardiac surgery included coronary artery disease, valvular heart disease, and aortic disease, with many patients also having comorbidities such as diabetes mellitus and cerebrovascular diseases. These similarities suggest that older men, often associated with habits like smoking and alcohol consumption, along with other unhealthy lifestyles, are more prone to cardiovascular and cerebrovascular diseases, which can compromise immune function and increase susceptibility to MDRO infections following hospitalization and surgical interventions.

A recent systematic review and meta-analysis conducted by Willems et al. on 44 studies involving a total of 14,049 patients from various medical settings colonized by MDR bacteria found a pooled cumulative incidence of infection of 14% at a median follow-up time of 30 days for MDR Gram-negative bacilli (GNB) and 8% at 30 days for vancomycin-resistant Enterococcus (VRE). However, the analysis did not specifically address patients who underwent surgery [20].

Peghin and colleagues conducted a study in September 2024 investigating the risk of infection following colonization with carbapenem-resistant Acinetobacter baumannii. However, they found no correlation or increased odds ratio between previous surgery and the development of infections in these patients [21].

When comparing our results with those obtained by De Pastena et al. [8], we found that pneumonia occurred in colonized patients in 3.7% of the cases in our study, whereas it occurred in 24.8% of the cases in their study. Similarly, the mortality rate was 3.7% in our study compared to 10% in theirs. Despite the similarities in our analyses, the results differed, which may be attributed to the differences in the types of surgery involved—both invasive, but significantly different in terms of the organs and sites affected. Moreover, it is important to note that in patients undergoing abdominal surgery, infections may be more frequent in the presence of preoperative colonization due to the nature of the surgery, which directly involves the gastrointestinal organs or is at least in close proximity to the site of colonization. Additionally, the variations in the postoperative management between our hospital and theirs could have contributed to these differences.

Mork et al. [5], for instance, evaluated last year the correlation between patient characteristics, operative variables, and the risk of bloodstream infection, as well as the association of primary bloodstream infection with adverse outcomes in cardiac surgery patients.

Our study is subject to several limitations. Firstly, its retrospective nature introduces inherent biases. Secondly, the relatively small sample size might have influenced our analysis, although the propensity score helped mitigate this by carefully selecting the control group. Lastly, we did not collect data on the duration of extracorporeal circulation, which could potentially impact the bloodstream diffusion of bacterial colonization.

However, considering the paucity of published data in the literature and the incidence of this (rare) condition among patients undergoing cardiac surgery, we truly believe this study remains a significant “hypothesis-generating” investigation that can be used as a basis for future in-depth research in this specific field.

## 6. Conclusions

In conclusion, this study did not find that rectal colonization by MDR worsens post-surgical outcomes in patients undergoing cardiac surgery. Despite the limitations of a retrospective study with a small patient sample, our findings support that MDR rectal colonization does not appear to increase adverse outcomes in cardiac surgery patients managed with standard in-hospital protocols. This observation aligns with the current prophylaxis guidelines, which do not differentiate based on the colonization status. However, this conclusion remains at the level of a hypothesis-generating study, and further prospective studies or confirmation by a larger retrospective registry is needed to confirm these results.

## Figures and Tables

**Table 1:** Analysis of propensity score in both groups of patients enrolled. BMI = body mass index; CABG = coronary artery bypass graft.

Positive Swab	Coefficient	Standard Error	Z	*p* Value *p* > |Z|	IC 95%
Age	−0.0114835	0.0113427	−1.01	0.311	−0.0337148 0.0107477
Sex	0.3043555	0.1766298	1.72	0.085	−0.0418325 0.6505434
BMI	−0.0987569	0.033217	−2.97	0.03	−0.163762 −0.0335537
Type of surgery (CABG vs. other)	0.331444	0.1707217	0.19	0.846	−0.3014639 0.3677528
EuroSCORE II	1.534036	1.266866	1.21	0.226	−0.9489756 4.017048

**Table 2:** Preoperative variable comparison in the unmatched cohort and in the matched cohort (control group) compared between the two groups. The values are expressed as mean ± standard deviation and number (percentage). BMI = body mass index; CABG = coronary artery bypass graft.

Unmatched Cohort	Rectal Swab − (n = 926)	Rectal Swab + (n = 27)	*p* Value
Age (years)	71.0 ± 6.7	69.6 ± 10.9	0.293
Sex (M/F)	470 (50.7%)/456 (49.3%)	18(66.7%)/19 (33.3%)	0.103
BMI (kg/m^2^)	29.0 ± 1.8	27.6 ± 4.9	0.001
CABG/other than CABG (n)	460 (49.7%)/466 (50.3%)	14 (51.8%)/13 (48.2%)	0.824
EuroSCORE II	3.2 ± 1.4	2.5 ± 1.1	0.010
Hypertension	918 (99.1%)	27 (100%)	0.628
Chronic kidney disease(eGFR < 30 mL/min)	221 (23.8%)	5 (18.5%)	0.584
Diabetes mellitus	715 (77.2%)	20 (74.1%)	0.702
Use of bronchodilators	95 (10.2%)	3 (11.1%)	0.886
Chronic steroid use	182 (19.6%)	4 (14.8%)	0.532
Chronic lung disease	98 (10.6%)	6 (22.2%)	0.056
Neurological disease	28 (3.0%)	1 (3.7%)	0.839
**Matched Cohort**	**Rectal Swab − (n = 27)**	**Rectal Swab + (n = 27)**	***p* Value **
Age (years)	68.3 ± 7.7	69.6 ± 10.9	0.700
Sex (M/F)	18(66.7%)/19 (33.3%)	18(66.7%)/19 (33.3%)	0.999
BMI (kg/m^2^)	27.8 ± 2.0	27.6 ± 4.9	0.778
CABG/other than CABG (n)	11 (40.7%)/16 (59.3%)	14 (51.8%)/13 (48.2%)	0.423
EuroSCORE II	2.6 ± 1.2	2.5 ± 1.1	0.751
Hypertension	27 (100%)	27 (100%)	0.999
Chronic kidney disease(eGFR < 30 mL/min)	6 (22.2%)	5 (18.5%)	0.735
Diabetes mellitus	18 (66.7%)	20 (74.1%)	0.551
Use of bronchodilators	4 (14.8%)	3 (11.1%)	0.685
Chronic steroid use	4 (14.8%)	4 (14.8%)	0.999
Chronic lung disease	5 (18.5%)	6 (22.2%)	0.735
Neurological disease	2 (7.4%)	1 (3.7%)	0.552

**Table 3:** Median preoperative NSQIP values in both groups. Values are expressed as median (IQR).

NSQIP Prevision	Rectal Swab − (n = 27)	Rectal Swab + (n = 27)	*p* Value
Severe complications (%)	16.8 (12.9–25.6)	17.7 (13.8–28.7)	0.412
Any complications (%)	22.9 (17.5–32.8)	23.5 (19.2–34.9)	0.539
Pneumonia (%)	4.8 (3.5–8.7)	5.0 (3.3–8.6)	0.667
Cardiac complications (%)	5.2 (4.1–7.8)	5.0 (4.0–7.4)	0.465
Surgical site infection (%)	3.0 (2.4–3.9)	3.1 (2.6–3.7)	0.509
Urinary tract infections (%)	2.0 (1.1–2.9)	2.1 (1.2–2.7)	0.701
Venous thromboembolism (%)	1.4 (0.8–1.9)	1.3 (0.9–1.8)	0.474
Renal failure (%)	4.0 (2.5–6.2)	4.1 (2.6–6.0)	0.807
Readmission (%)	10.8 (7.5–16.2)	11.2 (7.7–15.2)	0.715
Return to operating room (%)	4.1 (3.4–8.8)	4.3 (3.6–9.0)	0.366
Death (%)	4.5 (2.1–9.3)	4.6 (2.5–9.5)	0.865
Discharge to other structure (%)	24.8 (12.5–41.8)	26.7 (11.9–44.4)	0.531
Sepsis (%)	3.0 (2.0–4.8)	3.1 (2.1–5.1)	0.715

**Table 4:** Main postoperative outcomes. Values are expressed as median (IQR) and total number (percentage). ICU = intensive care unit.

Outcome Measure	Rectal Swab − (n = 27)	Rectal Swab + (n = 27)	*p* Value
ICU length of stay (days)	2 (1–4)	2 (2–4)	0.448
ICU readmission (n)	1 (3.7%)	4 (14.8%)	0.159
Hospital mortality (n)	1 (3.7%)	1 (3.7%)	0.999
Bacteremia (n)	1 (3.7%)	2 (7.4%)	0.552
Pneumonia (n)	1 (3.7%)	1 (3.7%)	0.999
Surgical site infection (n)	2 (7.4%)	6 (22.2%)	0.125
Urinary infection (n)	0 (0%)	0 (0%)	1
Gastrointestinal infection (n)	0 (0%)	0 (0%)	1

## Data Availability

The original contributions presented in the study are included in the article (and supplementary material), further inquiries can be directed to the corresponding authors.

## Supplementary Materials

The following supporting information can be downloaded at https://www.mdpi.com/article/10.3390/jcm13226897/s1, Table S1: Details of propensity score matching model, command “pstest”.

## References

[1] Mestrovic T., Robles Aguilar G., Swetschinski L.R., Ikuta K.S., Gray A.P., Davis Weaver N., Han C., Wool E.E., Hayoon A.G., Hay S.I. (2022). The burden of bacterial antimicrobial resistance in the WHO European region in 2019: A cross-country systematic analysis. Lancet Public Health.

[2] Nikaido H. (2009). Multidrug resistance in bacteria. Annu. Rev. Biochem..

[3] Parmanik A., Das S., Kar B., Bose A., Dwivedi G.R., Pandey M.M. (2022). Current Treatment Strategies Against Multidrug-Resistant Bacteria: A Review. Curr. Microbiol..

[4] Pacios O., Blasco L., Bleriot I., Fernandez-Garcia L., Bardanca M.G., Ambroa A., López M., Bou G., Tomás M. (2020). Strategies to Combat Multidrug-Resistant and Persistent Infectious Diseases. Antibiotics.

[5] Mork C., Gahl B., Eckstein F., Berdajs D.A. (2023). Prolonged cardiopulmonary bypass time as predictive factor for bloodstream infection. Heliyon.

[6] Wang Y.-C., Wu H.-Y., Luo C.-Y., Lin T.-W. (2019). Cardiopulmonary Bypass Time Predicts Early Postoperative Enterobacteriaceae Bloodstream Infection. Ann. Thorac. Surg..

[7] Sugawara G., Yokoyama Y., Ebata T., Igami T., Yamaguchi J., Mizuno T., Yagi T., Nagino M. (2018). Preoperative biliary colonization/infection caused by multidrug-resistant (MDR) pathogens in patients undergoing major hepatectomy with extrahepatic bile duct resection. Surgery.

[8] De Pastena M., Paiella S., Azzini A.M., Marchegiani G., Malleo G., Ciprani D., Mazzariol A., Secchettin E., Bonamini D., Gasparini C. (2018). Preoperative surveillance rectal swab is associated with an increased risk of infectious complications in pancreaticoduodenectomy and directs antimicrobial prophylaxis: An antibiotic stewardship strategy?. HPB.

[9] Righi E., Mutters N.T., Guirao X., del Toro M.D., Eckmann C., Friedrich A.W., Giannella M., Kluytmans J., Presterl E., Christaki E. (2023). ESCMID/EUCIC clinical practice guidelines on perioperative antibiotic prophylaxis in patients colonized by multidrug-resistant Gram-negative bacteria before surgery. Clin. Microbiol. Infect..

[10] Mazzeffi M., Gammie J., Taylor B., Cardillo S., Haldane-Lutterodt N., Amoroso A., Harris A., Thom K. (2017). Healthcare-Associated Infections in Cardiac Surgery Patients with Prolonged Intensive Care Unit Stay. Ann. Thorac. Surg..

[11] Healy D.G., Duignan E., Tolan M., Young V.K., O’connell B., McGovern E. (2011). Should cardiac surgery be delayed among carriers of methicillin-resistant Staphylococcus aureus to reduce methicillin-resistant Staphylococcus aureus-related morbidity by preoperative decolonisation?. Eur. J. Cardiothorac. Surg..

[12] Muñoz P., Hortal J., Giannella M., Barrio J., Rodríguez-Créixems M., Pérez M., Rincón C., Bouza E. (2008). Nasal carriage of S. aureus increases the risk of surgical site infection after major heart surgery. J. Hosp. Infect..

[13] Reddy S.L., Grayson A.D., Smith G., Warwick R., Chalmers J.A. (2007). Methicillin resistant Staphylococcus aureus infections following cardiac surgery: Incidence, impact and identifying adverse outcome traits. Eur. J. Cardiothorac. Surg..

[14] Garcia-Parejo Y., Gonzalez-Rubio J., Guerrero J.G., Sango A.G.-J., Escribano J.M.C., Najera A. (2024). Risk factors for colonisation by Multidrug-Resistant bacteria in critical care units. Intensive Crit. Care Nurs..

[15] Heath M.R., Fan W., Leu C.-S., Gomez-Simmonds A., Lodise T., Freedberg D.E. (2024). Gut colonization with multidrug resistant organisms in the intensive care unit: A systematic review and meta-analysis. Crit. Care.

[16] Bhatt P.J., Ali M., Rana M., Patel G., Sullivan T., Murphy J., Pinney S., Anyanwu A., Huprikar S., Taimur S. (2020). Infections due to multidrug-resistant organisms following heart transplantation: Epidemiology, microbiology, and outcomes. Transpl. Infect. Dis..

[17] Ren J., Duan S., Wu Y., Wen M., Zhang J., Liu Y., Zhu G. (2023). Multidrug-resistant bacterial infection in adult patients following cardiac surgery: Clinical characteristics and risk factors. BMC Cardiovasc. Disord.

[18] Jara M.C., Frediani A.V., Zehetmeyer F.K., Bruhn F.R.P., Müller M.R., Miller R.G., Nascente P.d.S. (2020). Multidrug-Resistant Hospital Bacteria: Epidemiological Factors and Susceptibility Profile. Microb. Drug Resist..

[19] Lorenzoni V.V., Rubert F.D.C., Rampelotto R.F., Hörner R. (2018). Increased antimicrobial resistance in Klebsiella pneumoniae from a University Hospital in Rio Grande do Sul, Brazil. Rev. Soc. Bras. Med. Trop..

[20] Willems R.P.J., van Dijk K., Vehreschild M.J.G.T., Biehl L.M., Ket J.C.F., Remmelzwaal S., E Vandenbroucke-Grauls C.M.J. (2023). Incidence of infection with multidrug-resistant Gram-negative bacteria and vancomycin-resistant enterococci in carriers: A systematic review and meta-regression analysis. Lancet Infect. Dis..

[21] Peghin M., Givone F., de Martino M., Ali R.W., Graziano E., Isola M., Grossi P.A. (2024). Risk factors for infection after carbapenem-resistant Acinetobacter baumannii colonization. Eur. J. Clin. Microbiol. Infect. Dis..

