# Supplementary material for: Role of Preoperative Multiple-Drug-Resistant Bacteria Intestinal Colonization in Cardiac Surgery: A Retrospective Study
Source: J Clin Med. 2024 Nov 16;13(22):6897. doi: 10.3390/jcm13226897 (PMC11594926; doi:10.3390/jcm13226897)
Supplement: Supplementary file 1 [file jcm-13-06897-s001.zip › jcm-3262323-supplementary.pdf]

Supplementary Table S1. Details of propensity score matching model, command "pstest".

Variable	Unmatched Matched	Mean treated	Mean control	% bias	% reduct |bias|	t test	P > |t|
**Age**	U	69.63	71.04	-15.6		-1.05	0.293
	M	69.63	68.63	11.0	29.4	0.29	0.700
**Sex_M**	U	0.667	0.507	32.4		1.63	0.103
	M	0.667	0.667	0.0	100	-0.00	1.000
**BMI**	U	27.59	29.04	-39.5		-3.87	0.001
	M	27.59	27.88	-7.8	80.2	-0.28	0.778
**Intervention**	U	0.518	0.496	4.3		0.22	0.824
	M	0.518	0.407	22.0	-410.7	0.81	0.423
**Euroscore**	U	2.510	3.205	46.5		1.92	0.010
	M	2.510	2.634	2.3	92.5	0.28	0.751

Sample	Ps R2	LR chi2	p>chi2	Mean bias	Med bias	Rubin's B	Rubin's R	% VAR
**Unmatched**	0.051	12.64	0.013	23.0	24.0	**53.2 (!)**	**4.26 (!)**	100
**Matched**	0.013	1.01	0.909	10.2	9.4	**21.9**	**1.74**	42

See PSMATCH2 and PSTEST commands in STATA for details.

For adequate matching: Rubin's B should be < 25%, Rubin's S should be between 0.5 and 2.
